# Ethnobotany of the Samburu of Mt. Nyiru, South Turkana, Kenya

**DOI:** 10.1186/1746-4269-2-35

**Published:** 2006-09-06

**Authors:** Rainer W Bussmann

**Affiliations:** 1 University of Hawaii, Lyon Arboretum, 3860 Manoa Rd., Honolulu, HI 96822, USA

## Abstract

Traditional plant use is of extremely high importance in many societies, and prevalent in African communities. This knowledge is however dwindling rapidly due to changes towards a more Western lifestyle. The influence of modern tourism cannot be neglected in this context.

This paper examines the plant use of the Samburu of the Mt. Nyiru area in Northern Kenya. The Samburu pastoralists of Kenya are still amongst the most traditional communities of the country and have retained most of their knowledge about the use of a large part of the plants in their environment for a wide variety of purposes.

The results indicate that the local population has a very high knowledge of the plants in their surroundings, and attributes a purpose to a large percentage of the plants found.

448 plant species were collected, identified and their Samburu names and traditional uses recorded. 199 species were reported as of "no use". The high proportion of 249 plant species however had some traditional use: The highest number (180 species) was used as fodder, followed by 80 species that had medicinal use. Firewood (59 species), construction (42 species), tools (31 species), food (29 species) and ceremonial use (19 species) ranked far behind.

Traditionally the Samburu attribute most illnesses to the effect of pollutants that block or inhibit digestion. This can include "polluted" food, contagion through sick people as well as witchcraft. In most cases the treatment of illness involves herbal purgatives to cleanse the patient. There are however frequent indications of plant use for common problems like wounds, parasites, body aches and burns.

The change from a nomadic to a more sedentary lifestyle, often observed in other areas of the country, has affected the Samburu of remote Mt. Nyiru to a much lesser extent and did so far not lead to a major loss of traditional plant knowledge. However, overgrazing and over-exploitation of plant resources have already led to a decline of the plant material available.

## Background

**Figure 1:**
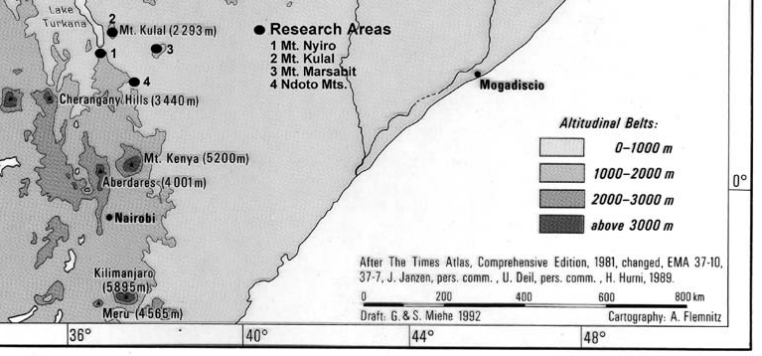
Study Area.

The Northern Region of Kenya (Fig. 1) occupies nearly 50% of the land surface area of the country, yet it has only received marginal biological attention. For a long time, basically only adventurers and big game hunters visited it. The colonial name "Northern Frontier District" clearly illustrates this situation.

It is still sparsely populated, and large parts are only accessible with difficulty. The few scientific studies that have been carried out in the North, mainly focused on its geological features [1-4]. The first, more comprehensive scientific research project, was the Unesco-IPAL study of the 1970's.

### Geology

The Northern Region consists of vast alluvial inland plains, inclining from altitudes of about 1200 m to the North of Mt. Kenya to barely 400 m around Lake Turkana. On the Southwest, a chain of mountains consisting of old crystalline Precambrian basement rocks, mainly extremely durable gneisses and granites, borders the plains. For this reason, the steep Ndoto and Nyiru Ranges, reaching up to 2752 m, were left standing during the different erosion cycles influencing the region [1,5]. Next to these, a series of Quarternary volcanic peaks, like Mt. Kulal (2285 m), Mt. Marsabit (1707 m) and the Huri Hills (1479 m), tower over the inland plains. In contrast to the soils of the plains, which consist mainly of Vertisols, Regosols, Lithosols and Cambisols, the mountain slopes are mainly covered with humic Acrisols over the basement formations, and deep, humic Andosols in volcanic areas [6-8].

### Climate

According to the climatological classification of Jätzold [9,10], the northern plains are part of the hot, arid tropical climate, with two short sub-humid seasons. Mean monthly temperatures range from 20–26°C in the plains, to 17–19°C in the mountains (Gatab, 1657 m). The average annual rainfall can be as low as 100–150 mm in the Hedad plain and Chalbi desert, rising to 500 mm in the valleys of the Nyiru and Ndoto mountains. In the mountain forest zone, a rainfall of about 1200 mm can be reached [11]. The main rainfall is concentrated in two wet seasons, from March to May and from October to December [12,13]. However, extreme rainfall occurs, e.g. 175 mm in 6 hours in Gatab on Mt. Kulal [8].

### Vegetation

Most mountain areas in Northern Kenya, located between 36°40'–38°00'E and 01°40'–03°40'N, are covered with evergreen montane forest. They owe their existence to the humidity received from mist condensation and frequent cloud formation in the peak areas. Neumann [14] after visiting the southern Turkana Region wrote: "The western face of Nyiru is.... topped with dark forests...., and here and there hang waterfalls...., filled with the outpourings of the heavy clouds which often cap the summit." Due to their enormous importance as water catchments [15], most mountain areas are gazetted as forest reserves. The extent of these reserves, however, does not really reflect the amount of land actually covered with forest. The Mt. Nyiru forest reserve measures a total of 45,496 ha of which barely 7,890 ha are covered with true forest [16].

The main reasons for forest destruction in the area are fires caused by honey-hunters and pastoralists, who burn the old grass at the start of the wet season. Others are overgrazing in the forest, and serving the firewood needs of a fast-growing population.

Despite their importance, few studies have been conducted on the montane forests of Northern Kenya. Beentje [16] gives some general remarks on the vegetation of the area and Synott [15] reports briefly on their status, importance and protection. Only Mount Kulal has received marginally more attention [18], and a plant checklist for this area was produced [19]. Based on 20 relevés on this mountain, Schultka & Hilger [20] distinguish mainly *Olea hochstetteri – Cassipourea malosana *and *Olea africana – Juniperus procera *forest.

Synott [15] reports that much less is known on the Nyiru and Ndoto forests than on the Kulal and Marsabit forests. He provides a checklist for the trees and shrubs of Mount Nyiru, containing 35 species. White [21] includes a short comment on the Marsabit region of Kenya and its afromontane forests, however mainly based on Synnotts observations from Mt. Kulal and Mt. Marsabit, without specific regard to Mt. Nyiro. Beentje [16] mentions that the vegetation of Mount Nyiru is "mostly unknown". Bytebier & Bussmann [22] provide the first detailed vegetation description and checklist of the Mt. Nyru flora, and Bussmann [23] gives an overview on the forest vegetation of all of Kenya's isolated smaller mountain ranges.

In the study presented here, the ethnnobotanical use of the forests of Mt. Nyiru by the local Samburu population was studied in greater detail.

### The Samburu

Plants have been an integral part of life in many indigenous communities, and Africa is no exception [24]. Apart from providing building materials, fodder, weapons and other commodities, plants are especially important as traditional medicines. Many tribes in Africa have a very elaborated plant knowledge [25]. Western influences have however led to an accelerating decline of this tradition. Most knowledge is still transferred entirely orally in many communities [26]. The "Witchcraft Act" of 1925 outlawed traditional medicine in Kenya. The practice however continued more concealed, until parts of the law were revoked with independence in 1963 [24]. Western style healthcare supplied by the government has been expanded in the last decades, but it is still often not readily available and many regions remain completely underserved. Subsequently, most communities still use herbal remedies as readily and cheaply available alternative. Although tourism has become a major income earner in Kenya, many local communities do not benefit from ths, and are rather further marginalized. The creation of game reserves has led to restrictions in the movements of nomatic communities like the Maasai and Samburu, and has restricted their grazing grounds [27-31].

The Samburu are mostly nomadic pastoralists, inhabiting the plains and highlands of Northern Kenya. Like their cousins the Maasai, they speak a "Maa" language and belong to the Chari-Nile branch of the Nilo-Saharan language family. Maa speaking peoples migrated into their current territory around the 16^th ^century [32]. The Samburu occupy some of the northernmost parts of Kenya, roughly between the town of Isiolo (on the northern slopes of Mt. Kenya), and Lake Turkana (Fig. 1). This territory has always been – and still is, one of the remotest accessible areas of Kenya, although the southern parts around the Buffalo Springs-Samburu Game Reserve complex have become a popular tourist destination in recent years. Due to this remote location the Samburu, unlike the Maasai, received very little attention from the colonial administration. Independence changed this only marginally. For this reason the traditional way of nomadic life is still much more prevalent amongst the Samburu, who migrate from their lowland wet season grazing grounds up to the humid mountain areas in the dry season [33].

Milk and blood from cows and soups derived from wild collected herbs are still the main pillars of Samburu diet. Especially children and women frequently eat berries and other wild fruits. Herbal knowledge is very widespread in the community, and many community members have an intricate knowledge of their environment.

Early accounts of Maasai plant use date back to the beginning of the last century [34,35] list already more than 400 herbal remedies used by the Maasai. A number of recent studies focus on the plant use of various sections of the Maasai [36,37]. Maasai history and customs [38] and migration [39-41] are well documented. Studies on Samburu Ethnobotany trace only back to the last two decades. Schultka & Hilger [20] and Kokwaro & Herlocker [42] provided some lists of Samburu plant use, while Heine et al. [43] compiled the most detailed lists of the plant use of Samburu from various sources. These studies concentrated however mostly on the lowland grazing grounds, and did not take the important highland forests sufficiently into account.

While most Bantu-speaking peoples in East Africa believe that illness is related to a curse from deceased ancestors, the Samburu attribute most illnesses to the effect of pollutants that block or inhibit digestion. This can include "polluted" food, contagion through sick people as well as witchcraft. In most cases the treatment of illness involves herbal purgatives to cleanse the patient. There are however frequent indications of plant use for common problems like wounds, parasites, body aches and burns [26,44].

Mayor health concerns include malaria, gastro-internal disorders, parasites, tuberculosis, brucellosis and Sexually Transmitted Diseases (STD's), while skin problems, burns, wounds and fractures are associated with the daily dangers of livestock keeping.

## Materials and methods

### Plant collections

The majority of plants were collected between 29 March 1995 and 2 April 1995 in the Collector Series Bytebier B., Mwangangi O.M., Kirika P., Waiganjo T., Newton M. & Bussmann R.W., abbreviated to Bytebier *et al*. All specimens were deposited at the East African Herbarium in Nairobi (EA), with duplicates at the Royal Botanic Gardens, Kew (K) and the National Botanic Garden of Belgium, Meise (BR). The author returned several times for additional vegetation ecological and ethnobotanical studies and his specimens (Collector Series Bussmann) are deposited at the Bayreuth University Herbarium. In addition to these collections, all specimens from Mt. Nyiro deposited in the East African Herbarium were taken into account for this study, comprising the following samples. Details are given in Additional File 1 (AF1) (see Table 6) and a few scattered collections leading to a total of 679 specimens, plus a few sight records by Bussmann RW. (SR in Additional File 1)

**Table 6:** 

Adamson J (AD in AF 1)	28 specimens	1947 & 1995/56
Archer PG (AR in AF 1)	20 specimens	1971
Bono G (BO in AF 1)	39 specimens	1977
Bussmann RW (BU in AF 1)	45 specimens	1995/96
Bytebier et al. (BY in AF 1)	325 specimens	1995
Cameron JBC (C in AF 1)	37 specimens	1972
Gilbert MG, Gachati FN & Gatheri GW (G in AF 1)	18 specimens	1978
Ichikawa M (I in AF 1)	5 specimens	1977
Kerfoot O (K in AF 1)	156 specimens	1960

### Nomenclature

The nomenclature of plant families follows Bamps [45], and in particular the available parts of the Flora of Tropical East Africa (FTEA) [46]. The nomenclature of genera and species of Pteridophytes, Monocotyledones and Dicotyledones follows the new edition of "Upland Kenya Wild Flowers" [47] The genus *Sinarundinaria *was treated according to Chao & Renvoize [48]. *Cyperaceae *and *Juncaceae *are named according to Haines & Lye [49], *Gramineae *according to Phillips [50]. The nomenclature of trees and shrubs is according to "Kenya Trees, Shrubs and Lianas" [51].

A total of 448 taxa belonging to 284 genera and 104 families are now on record. The families best represented were Asteraceae (36 species), Fabaceae (22 species), Graminae (20 species), Lamiaceae (19 species), Rubiaceae (15 species), Adiantaceae (14 species), Cyperaceae and Euphorbiaceae (13 species), Acanthaceae and Malvaceae (12 species) and Aspleniaceae (11 species) (Additional File 1).

### Ethnobotany

In order to get a more detailed inventory of plant use, ethnobotanical data were collected by interviews directly in the field during collection trips, and by discussing the freshly collected specimens with informants, after seeking oral consent from each respondent. This method was preferred over pure questionnaires to also get an indication for species that are not used by the community, and which are normally not mentioned during traditional interviews. All interviews were carried out with at least one local interpreter and assistant.

Data on plant species, families, vernacular names, parts used, traditional use and modality of use, as well as a comparison to plant use stated in [52] for the close-by Loita community were recorded and are given in Additional File 1.

### Results

#### Indigenous nomenclature

Heine et al. [43] and Heine [53] give an excellent overview on Samburu etymology and plant nomenclature. There are some differences in local dialect however.

Samburu plant nomenclature, as in many traditional societies, is very complex. Plant names are frequently related to plant appearance and use. It is very common that one vernacular name might refer to multiple species "*Mboroyo*" and "*Mboroyeyo" *for example refer to small Pteridophyta, "*Naigerigero*" to larger Pteridophyta, "*Segeet" *to Acanthaceae, "*Lanana*" generally to high value fodder grasses. In some cases Samburu terms reflect a specific use of a plant group, like "*Loperiai*", the name given to ceremonially important Cyperaceae, while "*Seiyei*" refers to Cyperaceae that are eaten by livestock, but do not have ceremonial significance. Some names are very specific to a genus. "*Ntulelei*" for example classifies all members of the genus *Solanum*.

However, in many cases Samburu plant nomenclature is much more exact, and many plants are referred to with a specific name, in particular if a plant has a specific use. In addition, names might indicate morphological characteristics or habitat. Since Samburu is originally not a written language, spelling of names is a matter of debate. The spelling used in this paper represents a consensus of the Samburu guides the author worked with, as well as the spelling suggestions of Heine et al. [43].

### Plant use

A total of 448 plant species belonging to 104 families were collected on Mt. Nyiru. Of these, 439 could be identified (Additional File 1). This represents a very large percentage of the total flora of the mountain region. The Samburu of Mt. Nyiru attributed a use to 249 species (56%) of the local flora. This number is very close to the compilation of Heine et al. [43] who presented a list of 580 species of plants found in the Eastern part of the Samburu range, 300 (52%) of which had some use.

The most important plant families for the Samburu were Asteraceae with 19 species used, Poaceae (18), Lamiaceae (15), Rubiaceae and Cyperaceae (13), Fabaceae (12) and Malvaceae (10). The different families have however a very different importance. Only about 50% of the total species in Asteraceae and Malvaceae were used, while virtually 100% of all species of the other plant families listed were used. In a very striking contrast to their cousins the Maasai, who use ferns for a wide variety of ceremonial purposes [37,52] the Samburu do not have any use for Pteridophyta.

An overview on the traditional use of plants on Mt. Nyiru in comparison to other areas of the country [37,43] is given in Additional file 1 and Table 1. The number of plant species found in the local flora is significantly higher in Eastern Samburu, with 580 species [43] in comparison to the 448 species found on Nyiru, and significantly lower in the Sekenani Maasai area in the South of the country [37]. This is due to a large contribution of lowland species in the East, and a complete lack of forest species in Sekenani. The percentage of species used in a specific use category show pronounced differences in the areas compared, an explanation for which is given in the respective category section.

### No use

The Samburu regarded 199 plant species found on Mt. Nyiru (44%) as "useless", and fifty-six of those did not carry any Samburu name. The most important families of "useless" plants were Asteraceae, Malvaceae, and all Pteridophyta, which contain a high percentage of herbal species, and have mostly no grazing value. This coincides very well with the observation that the Samburu, as many Maa speaking peoples, focus on the use of woody species, especially for medicines, and grasses (for fodder).

### Fodder

The single most important use of plants among the Samburu, unsurprising for a nomadic people, is fodder for livestock. A staggering 180 species (72% of all useful plants) was seen as useful as fodder. This figure comes very close to 69% of the useful species listed by Heine et al. [43] for that purpose. The Samburu show a clear distinction to the Maasai in this category. In Sekenani [37] only 33% of the useful species were used for fodder. This discrepancy can be explained by the fact that the Maasai of Sekenani are mostly using open grasslands as pastures, while the Samburu rely much more on forested areas as dry season grazing grounds.

### Medicinal use

Eighty (32%) of the useful plans found on Mt. Nyiru had medicinal properties. This falls very well in the range of other studies [37,43,52]. The most commonly used medicines treat ailments like malaria, fever, wounds, and stomach problems or are used for general strength (Table 2). Like in case of their cousins the Maasai, most Samburu medicines used to cure diseases have mainly the function of strong purgatives and emetics, in order to "cleanse" the body and digestive system from polluting substances. The frequent plant medicinal plant use by the Samburu for a large number of indications shows that governmental health care is hard to come by in this remote region of Kenya. When compared to other studies of Maasai and Samburu populations, remote areas feature in a very similar way [43,52], while populations close to health facilities have already lost a large part of their medicinal plant use [37].

**Table 2:** Uses of Medicinal Plants on Nyiru

	**Species used**	**Percentage**
**Malaria**	27	38
**Fever**	24	30
**Wounds**	23	30
**Soup (strength in adults)**	22	28
**Stomach problems**	16	20
**Sore throat**	10	13
**Toothbrush**	9	11
**Abortion/Exple placenta**	6	8
**Chest problems/Cough**	6	8
**Cough (children)**	5	6
**Eye problems**	5	6
**Tuberculosis**	5	6
**Gonorrhoea**	4	5
**Polio**	4	5
**Strength (children)**	4	10
**Joint and muscle pain**	3	4
**Cold**	2	3
**Parasites**	2	3
**Pneumonia**	2	5
**Pregnancy**	2	3
**Snakebite**	2	3
**Swollen breasts**	2	3
**Dysentery (Children)**	1	1
**Liver problems**	1	1
**Stomach (children)**	1	1

#### Malaria and fever

Malaria is very prevalent in many communities in Kenya [54], and the single most important condition treated with herbal remedies. Malaria and fever can be treated synonymously, although all informants mentioned both use categories consistently. 28 species (38%) of all medicinal plants were mentioned as treatment for malaria (30% for fever, 39% combined). Heine et al. [43] found a very similar number of species used for malaria in their study area. This indicates that the treatment of this disease is still a high priority for the Samburu, and that plant use is very consistent in the different areas. The treatment normally involves the ingestion of a boiled extract of the remedy to induce strong vomiting and diarrhea in order to purify the body. *Prunus africana *is used as especially potent malaria remedy. This species is already included in CITES Appendix II as endangered.

#### Wounds

Twenty-three species (30%) were used for the treatment of wounds. This represents more than twice the number of species found in other areas [43], indicating that wounds and wound infections are a very serious health threat in the Nyiru region. In most cases plants are either chewed, and the material is then applied to the wound as poultice (e.g. *Capparis tomentosa, Maerua angolensis, M. triphylla, Kalanchoe densiflora*), while *Crassula schimperi *and various species of *Hibicus *are burned and the ash applied to the wound.

The latex of *Gomphocarpus fruticosus, Commicarpus helenae *and *Euphorbia nyikae *is used to close wounds.

#### Stomach problems

Digestive problems and diarrhea are mainly treated with emetics, and most species of the 22 species (28%) used lead to strong vomiting and further diarrhea.

#### Soup (strengthening)

The ingestion of stews derived from a combination of herbs, boiled with milk and meat, is an important part of Samburu diet, and in particular common in warrior camps. 22 species (28% of the medicinal flora) are taken in this way to increase strength and stamina.

#### Cold

Common colds, sore throat and similar conditions are treated with 16 (20%) species. Either a decoction of various Commelinaceae is drunk to treat colds, or a decoction of Solanaceae is employed to gargle to treat a sore throat.

#### Pregnancy, menstruation and abortion

Complications in pregnancy and childbirth are potentially very dangerous conditions for women far from health care facilities. Samburu medicine focuses especially on the easement of birth, and to expel the placenta, employing for this purpose the same species that are otherwise used for abortions. Six plant species (*Leonotis nepetifolia, Abutilon hirtum, A. longicuspe, A. mauritianum, Acacia hookii, A. senegal*) serve this purpose. *Osyridicarpos schimperianus *is used to treat swollen breasts, while *Osyris abussinica *and *O. compressa *are burned and pregnant women are supposed to sit in the smoke to ease the pregnancy.

#### Chest problems and cough

The very smoky conditions in Samburu huts, where the smoke of the central fire escapes through a little window hole or the door, lead to a wide variety of chest and cough problems. Six plant species are used to ease these conditions in adults, five in children.

#### Toothbrush

The Samburu employ a variety (9 species, 11%) of fragrant shrubs as toothbrushes, chewing on a piece of cut branch to clean their teeth and to strengthen the gums.

#### Eye problems

Eye infections seemed to be very common in the Nyiru area. Eye problems were treated with 5 species. The juice of *Aloe *sp., *Vernonia galamensis *and *Ipomoea spathulata *was directly applied to the eye, while *Cleome *sp. and *Cleome usambarica *were burned, before applying the resulting ash on the eyes.

#### Tuberculosis, Polio

A variety of plants (*Aloe *sp., *Carissa edulis, Secamone punctulata, Myrsine africana, Rhamnus staddo, Cyphostemma bambuseti, C. kilimandscharicum*) were used to treat infections assessed as tuberculosis and polio.

#### Venereal diseases (Gonorrhea)

Venereal diseases were much less prevalent in Nyiru than in other study areas [43,52]. *Carissa edulis, Euphorbia heterochroma, Rhamnus staddo, Clerodendron myricoides *were employed as treatment.

#### Parasites

Worms and other intestinal parasites are a common feature for the Samburu. *Myrsine africana, Olea capensis *and *O. europaea *are used as strong anthelmintics. Western science has partly confirmed this use.

#### Snakebite

Although snakebites seem to be relatively rare, a few plants, namely *Rhamnus prinoides, Cardiospermum halicacabum *were employed to treat bites if they occurred.

#### Circulation and depression

The Samburu, who did not use any plants for this specific purpose, do not commonly share the "western" concept of circulatory problems and depression. It is however interesting to note that the Maasai of Sekenani, who are exposed to a continuous stream of tourism, and live relatively close to the provincial capital of Narok, have incorporated these concepts in their pharmacopeias [37].

### Parts of medicinal plants used and mode of application

Roots (41%) are the most frequently used plant parts, followed by leaves (25%), bark (19%), the juice and latex (7%), whole branches (6%, used as toothbrushes and splints) and fruits (2%) (Table 3).

**Table 3:** Plant part used for medicinal purposes

	**% of plant parts used**
**Root**	41
**Leaves**	25
**Bark**	19
**Juice/Latex**	7
**Branch**	6
**Fruit**	2

All remedies were prepared from fresh plant material, directly collected from the forest. No medicinal plant was cultivated.

Fifty percent of all medicinal plants were woody, while the remaining part represented herbs and grasses.

Diseases and other health problems were treated with single plants only. No decoctions of more than one species were used. However, if the patient did not improve, another plant of the same treatment category was applied. The plants were however frequently boiled with milk or meat to "give strength". When plants were used for "soup", any combination of the desirable species was added.

Of all preparations mentioned, plants were boiled (in water or milk and often with meat) in 68% of the cases. In 5% of the applications included plants soaked in water. (Table 4).

**Table 4:** Preparation methods for medicinal plants:

	**% of preparations**
**Boiled**	93
**Soaked**	7

In most cases this was done when the decoction was used for the treatment of children. In the remaining 27% of the application the plant or its latex was applied in natural form or burned and the ash applied, or the plant was chewed and applied as poultice, or just eaten.

The most frequent way to administer remedies was as to prepare a drink/stew and ingest this oral (64%), followed by poultice (10%, plant crushed and applied to wounds), 5% each were used as toothbrushes, eaten, burned and the ash applied to eyes and wounds, or a chewed gargled. 3% of the plants were squeezed and the juice or latex applied to wounds, eyes and in ears. 2% were used for whole body washes, 1% burned and the patient sat in the smoke, and 1% were used as splints for fractures (Table 5).

**Table 5:** Application method for medicinal plants on Nyiru

	**Number of species**
**Drink**	64
**Poultice**	10
**Toothbrush**	5
**Eat**	5
**Ash**	5
**Gargle**	5
**Juice in eye**	3
**Wash**	2
**Sit in smoke**	1
**Splint**	1

### Firewood

Firewood is still one of the most important commodities for many communities in the region and collected with great daily effort. 59 species (24% of the useful plants) were used for this purpose (25% in [43]). This is a striking contrast to the Sekenani Maasai region, where only 5% of all plants serve as firewood [37]. The Maasai have already shifted to Charcoal or Kerosene, easily available at small shops, for cooking purposes. The Samburu are not too eclectic with regard to species used as firewood. Hardwoods, or species selected for their pleasant smell are however preferred.

### Construction

Plant use for construction has traditionally a high significance, with 42 species (17%) used. Most plants in this category are used to frame traditional huts, or as fence for the living compounds.

### Tools

In the remote Nuiru region, a large number of plant species are used to make everyday tools like calabashes and headrests. Fragrant species, in particular many Acanthaceae, are used as mattresses. The most important "tool" are ropes and strings derived mostly from the bark of small trees, which are needed to tie the sticks used for traditional house construction together. 32 plant species (12%) were used for this purpose.

### Food

Use of plants as "food" means that in almost every case especially women and children eat the ripe fruits. 29 plant species (12%, in comparison to 26% given by Heine et al. 1988) were eaten, most importantly *Rhus natalensis, R. ruspolii, Carissa edulis, Capparis tomentosa, Dovyalis abyssinica, Turraea abyssinica, T. holstii, Rubus adolfi-friederici, R. apetalus, Vangueria apiculata, V. madagascariensis *and *V. volkensii*. Especially interesting was the use of mistletoe fruits (*Viscum triflorum *and *V. tuberculatum*) as food. Various other species (*Cyphostemma bambuseti, C. kilimandscharicum, Heteromorpha trifoliata*) were boiled as vegetables, and in two cases (*Kleinia odorata, Vigna schimperi*) the roots were eaten raw.

### Ceremonial

Ceremonial plant use has a very high importance in daily Samburu life. Many species have a specific ceremonial significance, mostly associated with blessings, age-rites and witchcraft. Nineteen species (8%) were used ceremonially. Olive (*Olea europaea*), is used in many ceremonies in the Maasai world [52], the Samburu did not attribute ceremonial power to Olive. In contrast, a large number of Cyperaceae was used as blessing of marriages, and Malvaceae were used as protective wristbands and necklaces. Ferns, which are extremely important in Maasai ceremonies [37,52], had absolutely no use in Samburu society.

### Arms

Carrying spears, swords and clubs (rungus), as well as sticks, is still very important in Samburu society in particular to indicate the position of a man in life. Spears are normally only carried by warriors (moran), while elders carry sticks. Clubs are virtually carried by every male, from small herding boys to ancients, while bows and arrows are more commonly seen in young boys. 17 species (5%) were used to manufacture arms. Straight branches of various *Acalypha *species (Euphorbiaceae) were preferred to make arrows. Meliaceae (*Tarenna abyssinica *and *T. holstii*) and Olives (*Olea capensis, O. europaea*) were the preferred species for rungus (warclubs), while the latter two species as well as *Dombeya goetzenii *and *D. rotundifolia *were in high demand for spear shafts. *Gnidia glauca *(Thymeleaceae) was the only species used as arrow poison. In this case the bark of the tree was boiled in water for several hours, and the remaining residue smeared on arrow tips.

### Bees

Honey plays a very important role in Samburu society, in particular as part of the dowry. Plant species attractive to bees are thus clearly singled out. Eight species were explicitly mentioned as "bee pasture", i.e. very important for honey production. Most of these (*Dicliptera laxata, Dyschoriste colorata, D. radicans, Hypoeste sforskahlii, H. triflora, Justicia lorata*) are Acanthaceae – a family that has almost no other use. A similar focus on plants for honey production was found amongst the Maasai [37], while Heine et al. [43] completely lack this category of plant use.

### Veterinary

The efficacy of plants used by the Samburu and Kikuyu for veterinary purposes was documented in recent studies [37,43,53,55-57]. Astonishingly, hardly any veterinary use of plants was encountered. Only 5 species (2%) of the useful plants were used as medicine for livestock. This trend can be observed in the same way in other studies [37,43,52] in different areas of the country. In striking contrast to medicinal care for people, governmental veterinary services seem to be much more available even in remote areas. In addition, the value attributed to livestock is traditionally so high that scarce resources were rather spent on caring for livestock, while traditional medicines were mostly used for human consumption.

### Beauty

Four species, most commonly *Justicia lorata, Dichrosephala chrysanthemifolia *and *D. integrifolia *were specifically used as perfume, normally with dried plant material used as necklace for good smell. This specific category was not encountered in other studies.

## Conclusion

Mt. Nyiru represents a very important dry season grazing area for the local Samburu population and studies on Samburu plant use have so far not focused on this zone. Traditional plant knowledge and use has still a high importance, and there are no indications that traditional knowledge has declined.

With the changes in lifestyle, more and more pronounced droughts, increasing population and the associated decline of the importance of plants, it is to be feared that Samburu ethnobotanical knowledge might get considerably limited however or disappear in the foreseeable future. This is the more evident since this knowledge is still mostly taught orally, without written record. An illustrated identification guide for Samburu plant use, best produced in Samburu or Kiswahili is long overdue. The local Samburu are however owners of this traditional knowledge, and any possible benefit resulting from its use has to be given to their community.

## Declaration of competing interests

The author(s) declare that they have no competing interests.

## Authors' contributions

All fieldwork, data analysis and compilation of this manuscript was conducted by the author.

**Table 1:** Plant uses by the Nyiru Samburu in comparison to [37] and [43]

**Plant uses**	**Species used (Nyiru)**	**Species used Nyiru (%)**	**Species used [43]**	**Species used % [43]**	**Species used [37])**	**Species used % [37]**
**No use**	199	44	280	48	51	33
**Fodder**	180	72	207	69	52	33
**Medicinal**	80	32	126	42	39	25
**Firewood**	59	24	75	25	7	5
**Construction**	42	17	78	26	17	11
**Tools**	31	12	78	14	6	4
**Food**	29	12	42	26	14	9
**Ceremonial**	19	8	39	13	21	14
**Arms**	17	7	0	0	8	5
**Bees**	8	3	0	0	4	3
**Veterinary**	5	2	0	0	5	3
**Beauty**	4	5	0	0	0	0

## Supplementary Material

Additional File 1 Species encountered and used on Mt. Nyiru. Indigenous names by Heine et al. [43] in parenthesis if differing from names found in this study. The data provided represent the complete overview on all plants encountered: Scientific names, vernacular names, uses. Click here for file
