# Supplementary material for: Ethnobotany of the Samburu of Mt. Nyiru, South Turkana, Kenya
Source: J Ethnobiol Ethnomed. 2006 Sep 6;2:35. doi: 10.1186/1746-4269-2-35 (PMC1570449; doi:10.1186/1746-4269-2-35)
Supplement: Additional File 1 — Species encountered and used on Mt. Nyiru. Indigenous names by Heine et al. [43] in parenthesis if differing from names found in this study. The data provided represent the complete overview on all plants encountered: Scientific names, vernacular names, uses. [file 1746-4269-2-35-S1.pdf]

Additional File 1.
Species encountered and used on Mt. Nyiru. Indigenous names by Heine et al. [43] in parenthesis if differing from names found in this study

Family/Genus	Species	Indigenous name	Use	Collection #
ACANTHACEAE				
Acanthopale	*pubescens* C.B. Clarke	Ltikilte (Ltikeinte)	no use	K2067
Crossandra	*massaica* Mildbr.	Seeget	no use	BY323
Dicliptera	*laxata* C.B. Clarke	Moten	1. bees, 2. for mattresses	K2068
Dyschoriste	*colorata* C.B. Clarke	Moten	1. bees, 2. for mattresses	BU9786
Dyschoriste	*radicans* Nees	Moten	1. bees, 2. for mattresses	BY189
Hypoestes	*forskahlii* (Vahl) R. Br.	Segeet , Maasai	1. eaten by animals and rarely by livestock, 2. bees	BY269; K2001
Hypoestes	*triflora* (Forssk.) Roem. & Schult.	Maasai	1. eaten by animals and rarely by livestock, 2. bees	BY210; K2076
Isoglossa	*gregorii* (S. Moore) Lindau	Siaiti	1. eaten by livestock	BY58; K2069
Justicia	*glabra* Koen. ex Roxb.	Segeet	1. eaten by livestock, 2. for mattresses	BY312
Justicia	*lorata* Ensermu	Segeet (Segheet)	1. bees, 2. eaten by livestock, 3. worn as necklace for good smell, 4. for mattresses	BY341, 350
Justicia	*striata* (Kl.) Bullock	Segeet	1. eaten by livestock, 2. for mattresses	BUSR
Thunbergia	*alata* Bojer ex Sims	Dakat (Sapau)	no use	BY258; K2090
ACTINIOPTERIDACEAE				
Actiniopteris	*semiflabellata* Pic. Serm.	Mboroiyo (Poroo)	no use	BY344
ADIANTACEAE				
Adiantum	*capillus-veneris* L.	Mboroiyo (Loigeigero)	no use	AR689; BY314; C0.151; G5209
Adiantum	*poiretii* Wikstr.	Mboroiyeyo	no use	BY76
Cheilantes	*multifidua* (Sw.) Sw.	Nkabariti	no use	BY253
Cheilanthes	*bergiana* Kuntze	Mboroiyeyo	no use	BU9811
Cheilanthes	*farinosa* (Forssk.) Kaulf.	Mboroiyeyo	no use	BU9809
Cheilanthes	*hirta* Sw.	Mboroiyeyo	no use	BU9812
Cheilanthes	*marantae* (L.) Domin	Mboroiyeyo	no use	C0.148
Cheilanthes	*multifida* (Sw.) Sw.	Mboroiyeyo	no use	BU9818; BY329,336; G5196
Cheilanthes	*tecta F.M.* Jarret ined.	Mboroiyeyo	no use	BU SR

Family/Genus	Species	Indigenous name	Use	Collection #
Cheilanthes	*farinosa*(Forssk.) Kaulf.	Naigerigero	no use	BY252
Doriopteris	*kirkii* (Hook.) Alston	Mboroiyeyo	no use	BU9752
Pellaea	*adiantoides* (Willd.) J.Sm.	Mboroiyeyo	no use	BU9797
Pellaea	*calomelanos* (Sw.) Link.	Mboroiyeyo	no use	BU9822; BY339
Pellaea	*longipilosa* Bonap	Mboroiyeyo	no use	BY327
ALOACEAE				
Aloe	sp.	Sukurwoi (Sukurwoi)	1. eye problems, squeeze and put juice in eye; 2. tuberculosis, boil for soup	BY360
AMARANTHACEAE				
Achyranthes	*aspera* L.	Lorokare (Lorokare)	1. eaten by livestock, 2. malaria, boil leaves in water	BY44
Celosia	*anthelminthica* Asch.	Ladum (Letamesi)	1. eaten by camels	K2024
Celosia	*schweinfurthiana* Schinz.	Ladum (Letamesi)	1. eaten by camels	BY309
Cyathula	*polycephala* Baker	Sulubei	1. eaten by animals and rarely by livestock	BY66
Pupalia	*lappacea* (L.) A. Juss.	Loreperepe (Lorepirepii)	1. esten by livestock	BY248
Sericocomopsis	*hildebrandtii* Schinz	Lturkan (Lturkwan, Lturkan)	1. eaten by livestock, 2. malaria, boil root and drink to make vomit, 3. stomach, crush and soak, drink to vomit	K2080
AMARYLLIDACEAE				
Scadoxus	*multiflorus* (Martyn) Raf.	Loilei	1. eaten by animals and rarely by livestock	BY256
ANACARDIACEAE				
Rhus	*natalensis* Krauss	Misigiyoi , Lmishkiyei (Lmiehkiyei)	1.leaves eaten by livestock, 2. fruit edible, 3. stomach, chew bark, 4. stomach children, crush leaves and root, soak in water, drink	BY278; K1948, 1949, 1950
Rhus	*ruspolii* Engler	Lmugurusian	1.leaves eaten by livestock, 2. fruit edible, 3. stomach, chew bark, 4. stomach children, crush leaves and root, soak in water, drink	BY276; K1945

Family/Genus	Species	Indigenous name	Use	Collection #
APIACEAE				
Alepidea	*peduncularis* A. Rich.	Lokowadii	no use	A539; AR688; BY206; C0.125; K2064
Anthriscus	*sylvestris* (L.) Hoffm. var. *sylvestris*	Lesirieni, Loisirieni	no use	BY209, 45
Ferula	*communis* L.	unknown	no use	K2026
Heteromorpha	*trifoliata* (H.L. Wendl.) Eckl. & Zeyh.	Lmasei (Lkunyeeni, Lkuyanoi, Lkuyeinei)	1. vegetable, boil and eat, 2. ceremonial, burn and use charcoal for protection	B21; BY291; K2027, 2028
Sanicula	*elata* Buch.-Ham. ex D. Don.	Loraborabi (Lerobic)	no use	BY18
Torilis	*arvensis* (Huds.) Link.	Laisirieni	no use	BU9784; BY264
APOCYNACEAE				
Carissa	*edulis* (Forssk.) Vahl.	Lamuriei (Lamuriei, Lamuriai)	1. polio, boil crushed root, drink, 2. gonorrhea, boil root, drink with milk, 3. malaria, boil crushed leaves, drink, 4. fences, 5. firewood, 6. fruit edible	K2023
ARALIACEAE				
Cussonia	*holstii* Engl. var. *holstii*	Guiyapiyapi (Loyapiyapi, Lbolorio, Lbolor)	1. leaves eaten by livestock, 2. strength, boil root for soup	B232; BY262; K1942
Schefflera	*volkensii* (Engl.) Harms	unknown	no use	BUSR
ASCLEPIADACEAE				
Ceropegia	*ballyana* Bullock	Garamurami	no use	BY249
Ceropegia	Sp	Loiyeti	no use	BY201
Cynanchum	*altiscandens* K. Schum.	Ilii	no use	BY263
Gomphocarpus	*fruticosus* (L.) W.T. Aiton	Lekule (Ltaguleti)	1. wounds, put latex on wound	BY240; K1979
Pergularia	*daemia* (Forssk.) Blatt. & MacOwan	unknown	no use	B33; K2021
Periploca	*linearifolia* Quart.-Dill. & A. Rich.	unknown	no use	K1998
Secamone	*punctulata* Decne	Sukurututi (Lekrra)	1. malaria, fever, boil root and drink, 2. stomach, boil root as tea, 3. infections, tuberculosis, boil root in soup	BY294, 363

Family/Genus	Species	Indigenous name	Use	Collection #
ASPARAGACEAE				
Asparagus	*africanus* Lam.	Lomeei (Lumei)	1. eaten by goats, 2. thread for calabashes	BY183
Asparagus	*falcatus* L. var. *ternifolius*	Lomei	1. eaten by goats, 2. thread for calabashes	BY270
ASPHODELACEAE				
Bulbine	*abyssinica* A. Rich.	Sogotiai, Kitampa	no use	BU9820; BY219, 151
ASPLENIACEAE				
Asplenium	*abyssinicum* Fee	Mboroiyeyo	no use	C0.149
Asplenium	*adiantum-nigrum* L.	Mboroiyeyo	no use	C0.133
Asplenium	*aethiopicum* (Burm f.) Bech.	Mboroiyeyo (Naigerigero)	no use	BU9698; BY72; C0.148; G5193
Asplenium	*elliottii* C.H. Wright	Mboroiyeyo (Naigerigero)	no use	BU9701; BY17A
Asplenium	*erectum* Willd. var. *usambarensis* (Hieron) Schelpe.	Mboroiyeyo (Naigerigero)	no use	BU9708; BY38, 251
Asplenium	*friesiorum* C. Chr.	Mboroyeiyo	no use	BU9715; BY6, 17B; C0.144
Asplenium	*loxoscaphoides* Baker	Mboroyeiyo (Naigerigero)	no use	BU9740
Asplenium	*monanthes* L.	Mboroiyeyo	no use	BU9704; BY73; C0.137
Asplenium	*strangeanum* Pic. Serm.	Negerigero, Mboroyeyo	no use	BY250; C0.136
Asplenium	*theciferum* (Kunth.) Mett.	Mboroiyeyo	no use	BU9706; BY74; C0.134
Asplenium	*trichomanes* L.	Mboroiyeyo	no use	BU9710; Co.135; G5195
ASTERACEAE				
Adenostemma	*perrottetii* DC.	unknown	no use	G5210; K2056
Berkheya	*spekeana* Oliv.	unknown	no use	AR705
Bidens	*flagellata* (Sherff) Mesfin	Loriapasei	1. eaten by livestock	BY213
Bidens	*hildebrandtii* O. Hoffm.	Lorubat	1. eaten by livestock	BY317
Bidens	*kilimandscharica* (O. Hoffm.) Sherff	Lorubat	1. eaten by livestock	K2057

Family/Genus	Species	Indigenous name	Use	Collection #
Bothriocline	*longipes* (Oliv. & Hiern.) N.E.Br.	Sunoni	1. eaten by livestock, 2. construction, 3. firewood, 4. fever, boil, drink and wash body, 5. strength and hygiene of babies, boil and wash, 6. malaria, boil and drink to make vomit	BY20; K2049
Carduus	*nyassanus* (S. Moore) R.E. Fr.	Sertet	no use	BY232
Cineraria	*deltoidea* Sond.	unknown	no use	K2046
Conyza	*newii* Oliv. & Hiern.	Lamiriruaki (Lamerrurrakii, Lamurrurrakii)	1. eaten by livestock	BY19; K2043
Conyza	*steudelii* Sch.-Bip. ex A. Rich.	Nasogoyo (Nasunkoyo e ldonyo)	1. eaten by livestock	BY224
Conyza	*stricta* Willd.	Nasogoyo, Songoyo (Nasunkoyo e ldonyo)	1. eaten by livestock	BY22, 83, 197; K2045
Conyza	*sumatrensis* (Retz.) E. Walker	Nasogoya Nasunkoyo e ldonyo)	1. eaten by livestock	BY225
Crassocephalum	*montuosum* (S. Moore) Milne-Redh.	Nasogoya (Sawaan)	no use	BUSR
Dichrocephala	*chrysanthemifolia* (Blume) DC.	Nasogoyo (Nypoorte)	1. eaten by livestock, 2. perfume	K2052
Dichrocephala	*integrifolia* (L.f.) Kuntze	Nasogoyo	1. eaten by livestock, 2. perfume	BY35
Emilia	*discifolia* (Oliv.) C. Jeffrey	Lukurijiji	no use	BY245; G5198; K2055
Emilia	*somalensis* (S. Moore) C. Jeffrey	Lukuruti	no use	BY80
Gerbera	*viridifolia* (DC.) Sch.-Bip.	Arapuraba	no use	BY178
Gnaphalium	*rubriflorum* Hilliard	Karamaite, Mutama	no use	BY226, 179
Gutenbergia	*cordifolia* Benth. ex Oliv.	Ludwaporo (Lodwporo)	no use, poisonous to cattle	BY287
Helichrysum	*argyranthum* O. Hoffm.	Kabiranabu	no use	A564
Helichrysum	*forskahlii* (J.F. Gmel.) Hilliard & B.L. Burtt var. *forskahlii*	Lokitegi, Sugoyo	no use	BY62,87
Helichrysum	*kilimanjari* Oliv.	Sulubei	1. eaten by livestock	BY26
Helichrysum	*nudifolium* (L.) Less. var. *nudifolium*	Lekole	no use	AR702; BY204

Family/Genus	Species	Indigenous name	Use	Collection #
Helichrysum	*odoratissimum* (L.) Less.	Sulubei	1. eaten by livestock	BU9759
Hirpicium	*diffusum* (O. Hoffm.) Roessler	Kunyanto	no use	BY239, 362
Kleinia	*odora* (Forssk.) DC.	Loinwaroi (Loinwaaroi, Loinwaaro)	1. eaten by livestock, 2. root eaten	G5187
Laggera	*elatior* R.E. Fr.	Lamuran	no use	57
Microglossa	*pyrifolia* (Lam.) Kuntze	Siaiti (Somati)	1. eaten by animals and rarely by livestock	BY8; K2047
Mikaniopsis	*bambuseti* (R.E. Fr.) C. Jeffrey	unknown	no use	BUSR
Osteospermum	*vaillantii* (Decne.) Norl.	unknown	no use	K2054
Senecio	*hadiensis* Forssk.	unknown	no use	B330
Senecio	*syringifolius* O. Hoffm.	Loiyeti	1. eaten by livestock	BY180
Sonchus	*afromontanus* R.E. Fr.	Saatia, Sweta	1. eaten by animals and rarely by livestock	BY168, 216
Vernonia	*galamensis* (Cass.) Less. ssp. *nairobiensis* M.G. Gilbert	Sojaa (Sojaa)	1. eye problems, squeeze juice in eye	BY196
Vernonia	*hymenolepis* A. Rich.	Leamarat	no use	B20; K2041
Vernonia	*syringifolia* O. Hoffm.	Leamarat, Siati	no use	BY169, 9
BALSAMIACEAE				
Impatiens	*hochstetteri* Warb.	Mosai	no use	BU9785
Impatiens	*meruensis* Gilg	Mosai	no use	C0.138
Impatiens	*meruensis* Gilg ssp. *septentrionalis* Grey-Wilson	Mosai	no use	BY51; K2082
Impatiens	*sodenii* Engl.	Iljesi	no use	ATG; B202; BU9783; BY325; K2083
BEGONIACEAE				
Begonia	sp.	unknown	no use	BY324
BERBERIDACEAE				
Berberis	*holstii* Engl.	unknown	no use	K1939
BORAGINACEAE				
Cordia	*monoica* Roxb.	Lgweita	1. eaten by livestock,2. fruit eaten, 3. construction, fences, 4. firewood, 5. fractures, use as splint, 6. ceremonial	B26; K1938

Family/Genus	Species	Indigenous name	Use	Collection #
Cynoglossum	*coeruleum* A. DC.	Loreperepe, Saali (Saali)	no use	BY41, 176
Cynoglossum	*coeruleum* A. DC. ssp. j*ohnstonii* (Baker) Verdc.	Saali	no use	K2095
Lithospermum	*afromontanum* Weim.	unknown	no use	K2006
BRASSICACEAE				
Arabis	*glabra* (L.) Bernh.	Seri	no use	BY175
Cardamine	*africana* L.	Losayioi (Losayioi)	no use	AR690
CAESALPINIACEAE				
Chamaecrista	*usambarensis* (Taub.) Standley	Dabibi	no use	A565; BY199
Crotalaria	*fascicularis* Polhill	unknown	no use	B28
Crotalaria	*incana* L. ssp. *purpurascens* (Lam.) Milne-Redh.	Sag'elg'eli, Ladarara (Letuala, Naitarrei, Ladarara)	1. strength, boil bark with tea, 2. coughs and chest pain, chew bark and spit	BY152, 265
Crotalaria	*keniensis* Baker f.	Lekelden	no use	K1913
Crotalaria	*lachnocarpoides* Engl.	Nterepirepi	no use	BY156; K1906, 1907
Crotalaria	*natalitia* Meisn. var. *natalitia*	Lantarara	1. strength, boil bark with tea, 2. coughs and chest pain, chew bark and spit	B132; BY274; K1914
Crotalaria	sp.	Lekelden	no use	BY212
Pterolobium	*stellatum* (Forssk.) Brenan	unknown	no use	K1935
CALLITRICHACEAE				
Callitriche	*stagnalis* Scop.	Maasai (Masai)	1. eaten by animals and rarely by livestock	B208; BY231
CAMPANULACEAE				
Campanula	*edulis* Forssk.	unknown	no use	AR695; K2097
Wahlenbergia	*abyssinica (*A. Rich.) Thulin ssp. *abyssinica*	Kabiranabu	no use	A541; BU9778; BY92; C0.108; K2063
Wahlenbergia	*capillacea* (L.f.) A.DC. ssp. *tenuior* (Engl.) Thulin	Ateptep	no use	BY93
Wahlenbergia	*lobelioides* (L.f.) A.DC. ssp. *nutabunda* (Guss.) Mirb.	unknown	no use	AR697
Wahlenbergia	*virgata* Engl.	Lamuran, Lonyori, Pesho	no use	A540; AR694; BY77

Family/Genus	Species	Indigenous name	Use	Collection #
CAPPARACEAE				
Capparis	*tomentosa* Lam.	Laturdei (Loturdei)	1. eaten by livestock, 2. fruit eaten, 3. wounds and burns, chew leaves and put on wound, 4. construction and fences	BY334; K1985
Cleome	sp.	Lkitagisii	1. eaten by livestock, 2. roots for soup, 3. eye problems, burn and put ash in eye	K2062
Cleome	*usambarica* Pax	Lkitagisii (Loiburke, Loiborkwe)	1. eaten by livestock, 2. roots for soup, 3. eye problems, burn and put ash in eye	BY318
Crateva	*adansonii* DC.	unknown	no use	K1990
Gynandropsis	*gynandra* (L.) Briq.	Loikat (Lasayet, Lasayyet)	1. to make containers	BY288
Maerua	*angolensis* DC.	Loitaakine (Loitakine)	1. eaten by livestock, 2. construction, 3. firewood, 4. wounds, chew plant and apply, 5. for containers	K1931
Maerua	*triphylla* A. Rich.	Loitaakine (Mulingila)	1. eaten by livestock, 2. construction, 3. firewood, 4. wounds, chew plant and apply, 5. for containers	BY345
Thylachium	*africanum* Lour.	unknown (Lolmugi, Sankaratei)	no use	K1958
CARYOPHYLLACEAE				
Cerastium	*indicum* Wight & Arn.	Impian	no use	BY164; K2073
Drymaria	*cordata* (L.) Willd. ex Roem. & Schult.	Llokii	1. eaten by livestock	BY1; K2096
Pollichia	*campestris* Aiton	Iljesii (Nkaakuji)	1. esten by livestock	BY238; K2099
Silene	*burchellii* Otth. ex DC.	Makarat	1. eaten by animals and rarely by livestock	A552
Silene	*macrosolen* Steud. ex A. Rich.	Makarat	1. eaten by animals and rarely by livestock	K2074

Family/Genus	Species	Indigenous name	Use	Collection #
Stellaria	*sennii* Chiov.	Maasai (Sarepirepi)	1. eaten by animals and rarely by livestock	BY215
CELASTRACEAEA				
Maytenus	*heterophylla* (Eckl. & Zeyh.) N. Robson	Saramunai (Sagumai)	1. eaten by livestock, 2. strength, boil for soup, 3. fruit eaten, 4. for smoking calabashes	BY279
Mystroxylon	*aethiopicum* (Thunb.) Loes.	Saramunai (Lodo nanayyoi, Saramunai)	1. firewood, 2. construction and fences	BY319
CHENOPODIACEAE				
Chenopodium	*schraderianum* Schult.	Lomugulale (Lapuyagi, Lapuyaki)	no use	BY289
COMMELINACEAE				
Aneilema	*leiocuale* K. Schum.	Naiteteiyai (Naiteteyyai, Naiteteyyei)	1. eaten by livestock, 2. cough in children, crush and boil with milk	BUSR
Commelina	*benghalensis* Wall.	Naiteteiyai (Naiteteyyai, Naiteteyyei, Nkaiteteyyai)	1. eaten by livestock, 2. cough in children, crush, boil with milk	K2092
Commelina	*africana* L.	Naiteteiyai (Naiteteyyai, Naiteteyyei, Nkaiteteyyai)	1. eaten by livestock, 2. cough in children, crush, boil with milk	BY154
Commelina	*foliacea* Chiov.	Naiteteiyai (Naiteteyyai, Naiteteyyei, Nkaiteteyyai)	1. eaten by livestock, 2. cough in children, crush, boil with milk	BY36
Cyanotis	*foecunda* Hassk.	Naiteteiyai	1. eaten by livestock, 2. cough in children, crush, boil with milk	BU9781; BY343
COMMIPHORACEAE				
Commiphora	sp.	Lekawa (Lekawap)	1. toothbrush	BY247
CONVOLVULACEAE				
Ipomoea	*spathulata* Hallier f.	Lokitegi	1. eye problems, squeeze and put juice in eye	BY300

Family/Genus	Species	Indigenous name	Use	Collection #
Ipomoea	*wightii* (Wall.) Choisy	Lagit (Lgisoyyai, Lkisoyyai, Llkiseyyai)	1. eaten by livestock	BY273
CRASSULACEAE				
Crassula	*alba* Forssk.	Gokudogit	no use	AR699; BY217
Crassula	*alsinoides* (Hook. f.) Engl.	unknown	no use	K2091
Crassula	*nodulosa* Schoenl. var. *nodulosa*	Loinonondero (Loinonondero)	no use	AR698; K2025
Crassula	*schimperi* Fisch. & C.A. Mey. ssp. *schimperi*	Lokumeki, Lorogon (Lokumeki, Lokimeki)	1. wounds, burn and put ash on wound	BY85, 243
Kalanchoe	*citrina* Schweinf.	unknown (Lkicein, Losisanjo)	no use	BY340, 356; K2086
Kalanchoe	*densiflora* Rolfe var. *densflora*	Lekarate (Lekarate)	1. stomach, boil for soup, 2. wounds, chew and apply	BY220; K2087
CUCURBITACEAE				
Coccinia	*sp.*	Sogoroi (Loisirarui)	no use	BY255
Gerrardanthus	*lobatus* (Cogn.) C. Jeffrey	Garaurau	no use	BY307
Lagenaria	*abyssinica* (Hook. f.) C. Jeffrey	Lagitok (Leakitok)	1. eaten by monkeys	BY52
Lagenaria	sp	Lagitok (Leakitok)	1. eaten by monkeys	BY355
CUPRESSACEAE				
Juniperus	*procera* Endl.	Tarakye, Ntarakwai (Ltarakwai)	1. eaten by livestock, 2. for mattresses, 3. chew fruit for sore throat	A393,6162; BY188
CYPERACEAE				
Cyperus	*comosipes* Mattf. & Kuek. ssp. *Comosipes*	Seiyei	1. eaten by livestock, 2. ceremonial, blessing of marriage, as protection, 3. fever, boil root and drink	BY257
Cyperus	*dichroostachyus* A. Rich.	Loperiai (Loperiai)	1. eaten by livestock, 2. ceremonial, blessing of marriage, as protection, 3. fever, boil root and drink	BY234; K1926
Cyperus	*impubes* Steud.	Seiyei	1. eaten by livestock, 2. ceremonial, blessing of marriage, as protection, 3. fever, boil root and drink	BY261

Family/Genus	Species	Indigenous name	Use	Collection #
Cyperus	*niveus* Retz. var. *leucocephalus* (Kunth) Fossberg	Loperiai	1. eaten by livestock, 2. ceremonial, blessing of marriage, as protection, 3. fever, boil root and drink	K1924
Cyperus	*rigidifolius* Steud.	Loperai, Seiyai	1. eaten by livestock, 2. ceremonial, blessing of marriage, as protection, 3. fever, boil root and drink	BY59, 159
Cyperus	*rohlfsii* Bieck.	Seiyai	1. eaten by livestock, 2. ceremonial, blessing of marriage, as protection, 3. fever, boil root and drink	BY342
Cyperus	*sesquiflorus* (Torr.) Mattf. & Kuek. ssp. *appendiculatus* (K. Schum.) Lye	Loroki	1. eaten by livestock, 2. ceremonial, blessing of marriage, as protection, 3. fever, boil root and drink	BY71
Cyperus	sp.	Seiyai	1. eaten by livestock, 2. ceremonial, blessing of marriage, as protection, 3. fever, boil root and drink	BY68
Cyperus	sp.	Seiyai	1. eaten by livestock, 2. cerem. blessing of marriage, as protect., 3. fever, boil root drink	BY160
Cyperus	sp.	Seiyei	1. eaten by livestock, 2. ceremonial, blessing of marriage, as protection, 3. fever, boil root and drink	BY246
Mariscus	sp.	Masuti (Lmasuuti)	1. eaten by livestock	BY177
Pycreus	*niger* Ruiz & Pav. ssp. *elegantulus* (Steud.) Lye.	Loperiai	1. eaten by livestock, 2. ceremonial, blessing of marriage, as protection, 3. fever, boil root and drink	K1925
Schoenoxiphium	*lehmannii* (Nees) Steud	Seteti (Serteet)	1. eaten by livestock	BU9819
DENNSTAEDTIACEAE				
Hypolepis	*goetzei* Reimers	Naigerigero	no use	C0.139

Family/Genus	Species	Indigenous name	Use	Collection #
Pteridium	*aquilinum* (L.) Kuhn.	Naigerigero	no use	BY194
DIPSACACEAE				
Dipsacus	*pinnatifidus* A. Rich.	Lorugun	no use	AR704; BU9821; BY184; K2003
Scabiosa	*columbaria* L.	unknown	no use	A555; AR696; C0.123; K2075
Scabiosa	*columbaria* L.	unknown	no use	A555; AR696; C0.123; K2075
DRACAENACEAE				
Dracaena	*afromontana* Mildbr.	Lkaajo (Nkakidonit)	1. for making small containers	BY21
Dracaena	*ellenbeckiana* Engl.	Gokudogit (Nkakidonit)	1. for making small containers	BY358
DRYOPTERIDACEAE				
Arachniodes	*foliosa* (C.Chr.) Schelpe	Naigerigero	no use	C0.13
Polystichum	*fuscopaleaceum* Alston	Negerigero	no use	B23; BU9803, 9796; BY50; C0.147
ERICACEAE				
Agauria	*salicifolia* (Lam.) Oliv.	unknown	no use	K1970
Erica	*arborea* L.	Lemario	1. firewood	B214; BY171; C0.118; K2018
Erica	*mannii* (Hook. f.) Beentje ssp. *usambarensis* (Alm & T.C.E. Fr.) Beentje	Uali	1. firewood	BY187
EUPHORBIACEAE				
Acalypha	*fruticosa* Forssk. var. *euglandulosa* Radcl.-Sm.	Siati (Siaiti, Ibor)	1. eaten by livestock, 2. construction, 3. arrows	K1994
Acalypha	*fruticosa* Forssk. var. *fruticosa*	Siati (Siaiti, Ibor)	1. eaten by livestock, 2. construction, 3. arrows	B121
Acalypha	*volkensii* Pax	Gomei	1. eaten by livestock, 2. construction, 3. arrows	BY260; K1995
Bridelia	*micrantha* (Hochst.) Baill.	Loisiteti (Lapironit)	no use	I906; K1943
Clutia	*abyssinica* Jaub. & Spach. var. *abyssinica*	Logul	no use	B142; BY193

Family/Genus	Species	Indigenous name	Use	Collection #
Croton	*dichogamus* Pax	Lakirding'ai (Lakirdenai, Loiborbene, Nkitarru, Nakayyapiyapi)	1. malaria, boil root in water, drink, 2. stomach, boil root, drink, 3. chest problems, boil root, drink, 4. firewood, 5. construction	B27
Croton	*megalocarpus* Hutch.	Lmargweet (Lmaragweet, Lmarakweet)	1. eaten by livestock, 2. malaria, boil root in water, drink, 3. stomach, boil root, drink, 4. chest problems, boil root, drink, 5. firewood	K1961
Euphorbia	*brevicornu* Pax	Lpwoi	no use	BY56
Euphorbia	*depauperata* A. Rich. var. *depauperata*	Lorte	no use	BY170
Euphorbia	*heterochroma* Pax	Lkelelit (Ncelelit)	1. malaria, fever, boil in soup, 2. gonorrhoea, boil in soup, 3. chest problems, boil as tea	BY361
Euphorbia	*nyikae* Pax var. *nyikae*	Loparaa	1. malaria, boil in soup, 2. wounds and burns, put latex on wound	BY359
Phyllanthus	*fischeri* Pax	Lokirisai	1. eaten by livestock, 2. firewood	K2017
Phyllanthus	*sepialis* Muell.-Arg.	Lokirisai	1. eaten by livestock, 2. firewood	B122
FABACEAE				
Amphicarpa	*africana* (Hook. f.) Harms	unknown	no use	K1915
Argyrolobium	*fischeri* Thunb.	unknown	no use	AR703; B30; C0.113
Cadia	*purpurea* (Picc.) Aiton	Lkigiriai (Lgigiriai)	1. eaten by livestock, 2. firewood, 3. construction and fences, 4. against flees and parasites, crush, put in water, wash	BY333; I899; JBH69/51; JB11777; K1905
Desmodium	*repandum* (Vahl.) DC.	Ladakat (Ladakat orok)	1.eaten by livestock and wild animals, 2. construction	K1918

Family/Genus	Species	Indigenous name	Use	Collection #
Dolichos	*Sericeus* E. Mey. ssp. *sericeus*	Lmontoi (Lmontoi, Mentei)	no use	B320
Glycine	*wightii* (Wight & Arn.) ssp. *labialis* (A. Rich.) Verdc. var. *mearsii* (De Wild) Verdc.	unknown	no use	K1917; I905
Indigofera	*atriceps* Hook. f. ssp. *atriceps*	Imiim (Imiim)	1. eaten by livestock, 2. toothbrush, 3. cosmetic for blackening lips	K1909
Indigofera	*lupatana* Baker f.	Lobobo (Lobobo le tabasi)	1. eaten by animals and rarely by livestock	K1912
Indigofera	*schimperi* Jaub. & Spach. var. *schimperi*	unknown	no use	G5192
Indigofera	*swaziensis* Bolus var. *swaziensis*	unknown	no use	K1910
Indigofera	*volkensii* Taub.	Dabibi	no use	BY37; G5190A
Kotschya	*recurvifolia* (Taub.) F. White ssp. *keniensis* Verdc.	unknown	no use	C0.119
Lablab	*purpureus* (L.) Sweet ssp. *uncinatus* Verdc.	unknown	no use	K1922
Lotus	*goetzei* Harms	unknown	no use	K1911
Mucuna	*gigantea* (Willd.) DC. ssp. *quadrialata* (Baker) Verdc.	Larashi	no use	BY298
Ormocarpum	*trachycarpum* (Taub.) Harms	Lkigeria (Lkegweita, Lkekantei, Lkigeria)	1. eaten by livestock, 2. fences, 3. sticks, 4. fruit edible	K1937
Psoralea	*foliosa* Oliv.	unknown	no use	A537
Tephrosia	*interrupta* Engl. ssp. *interrupta*	Silikai (Silik)	1. eaten by animals and rarely by livestock	A559; B133; G5204; K1908
Teramnus	*labialis* (L.f.) Spreng. ssp. *labialis* var. *abyssinicus* (A. Rich.) Verdc.	unknown	no use	K1920
Trifolium	*semipilosum* Fresen. var. *semipilosum*	unknown	no use	B204
Vigna	*schimperi* Baker	Loiyeti	1. eaten by livestock, 2. root edible	BY34; K1916
Zornia	*setosa* Baker f. ssp. *obovata* (Baker f.) J. Leon & Milne-Redh.	Ragai	no use	BY91
**FLACOURTIACEAE**				
Dovyalis	*abyssinica* (A. Rich.) Warb.	Limoroo (Lumuroo, Lmoroo)	1. eaten by livestock, 2. fruit eaten, 3. firewood, 4. fences	BY158; K1965

Family/Genus	Species	Indigenous name	Use	Collection #
Trimeria	*grandifolia* (Burkill) Sleumer ssp. *tropica*	Ledat (Ledat)	1. malaria, boil plant and drink	B39
GERANIACEAE				
Geranium	*aculeolatum* Oliv.	Loitenenei	no use	K2038
Geranium	*arabicum* Forssk.	Loitenenei	no use	B201; BY190A; K2036
Pelargonium	*alchemilloides* (L.) Ait. f. ssp. *multibracteatum* (A. Rich.) Kokwaro	Logororeki (Letamesi)	1. eaten by animals and rarely by livestock	B205; BU9757; BY244
Pelargonium	*whytei* Baker	Logororeki	1. eaten by animals and rarely by livestock	K2037
GUTTIFERAE				
Garcinia	*livingstonei* T. Anderson	Lgarsoyi (Lgarsoyi)	1. firewood	BY337
Hypericum	*kiboense* Oliv.	unknown	no use	A558
Hypericum	*revolutum* Vahl.	Supat	1. firewood	A562; BY191; C0.177; K1932
Hypericum	*roeperanum* A. Rich.	Supat	1. firewood	K1934
HALORAGACEAE				
Gunnera	*perpensa* L.	Larau	no use	BU9788; BY230
HYACINTHACEAE				
Albuca	*abyssinica* Jacq.	Naimarasui	1. eaten by wild animals	BY157
HYPOXIDACEAE				
Hypoxis	*kilimanjarica* Baker ssp. *kilimanjarica*	Ilkurotii	1. eaten by animals and rarely by livestock	BU9776; BY90
Hypoxis	*obtusa* Burch.	Siederi (Naingungnai)	1. eaten by animals and rarely by livestock	BY214
IRIDACEAE				
Aristea	*alata* Baker	Lara, Suoderi	1. eaten by animals and rarely by livestock	A535; BY79
Gladiolus	*goetzei* Harms	Bolorio	no use	AR701
Gladiolus	*newii* Bak.	Bolorio	no use	BY218
JUNCACEAE				
Juncus	*oxycarpus* Kunth.	Likwashe	no use	BU9760; BY81
LAMIACEAE				
Aeollanthus	*repens* Oliv	Sakurudumi	no use	BY259
Becium	*decumbens* (Guerke) A.J. Paton	Loisau	1. eaten by livestock	BY78

Family/Genus	Species	Indigenous name	Use	Collection #
Becium	*obovatum* (E. Mey. ex Benth.) N.E. Br.var. *capitatum*	Lorriambiti (Lorriambiti)	no use	AR700; K2094
Leonotis	*nepetifolia* (L.) R. Br.	Gishieni (Lkiceni, Lcicieni)	1. abortion, soak leaves in water, drink	BY60
Leucas	*grandis* Guerke	unknown	no use	G5214; K2089
Leucas	*urticifolia* (Vahl) R. Br. var. *annulata* Sebald	Lomasigarai (Lolpidipid, Lturle)	1. eaten by livestock	BY283
Ocimum	*suave* Willd.	Lemuran (Lomunyaani, Lominyani, Leminyaani)	1. eaten by livestock, 2. leaves smoked	BY281; K2002
Plectranthus	*barbatus* Andr.	Lbolan (Lolan, Lbalan)	1. eaten by livestock, 2. strength, boil with soup	K1992
Plectranthus	*edulis* (Vatke) Agnew	Lekeldei	1. eaten by livestock, 2. strength, boil with soup	BY233
Plectranthus	*grandicalyx* E.A. Bruce	Lokuruki	1. eaten by livestock, 2. strength, boil with soup	BY84
Plectranthus	*igniriaus* (Schweinf.) Agnew	Saalai (Saali)	1. eaten by livestock, 2. strength, boil with soup	G5216
Plectranthus	*sylvestris* Guerke	Laamarat (Saali le ntim)	1. eaten by livestock, 2. strength, boil with soup	BY70
Salvia	*nilotica* (Juss.) Jacq.)	Lamuran	no use	BY23; K2066
Satureia	*abyssinica* (Benth.) Briq.	Lekeneia (Lekeneyya)	1. eaten by livestock	AR691; B115; C0.112
Satureia	*biflora* (D. Don.) Benth.	Lanana (Lekeneyya)	1. eaten by livestock	BY181; C0.109; K2029
Satureia	*pseudosimensis* Brenan	Naitarakwai	1. eaten by livestock, 2. leaves for tea	BY205
Satureia	sp.	Saina	1. eaten by livestock	BY192
Satureia	sp.	Sunoni	1. eaten by livestock	BY29
Tinnea	*aethiopica* Kotschy ex Hook. f.	Lmanture (Logildia, Logolkeek, Lokildia)	1. eaten by livestock, 2. firewood	BY326
LINACEAE				
Linum	*keniense* T.C.E. Fr.	unknown	no use	BU9780
Linum	*volkensii* Engl.	Mani	no use	A547; BY95

Family/Genus	Species	Indigenous name	Use	Collection #
LOBELIACEAE				
Lobelia	*giberroa* Hemsl.	Lekeldei	no use	BY236
Lobelia	*holstii* Engl.	Segeet	1. eaten by livestock, 2. for mattresses	A566; BY207; C0.124; K2085
Monopsis	*stellarioides* (C. Presl.) Urb. ssp. *schimperiana* (Urb.) Thulin	Maasai	no use	BY202
LOGANIACEAE				
Nuxia	*congesta* Fresen.	Lgotoi (Ntapukai, Lmusuncak)	1. eaten by livestock, 2. fruit edible, 3. firewood, 4. fences	B25; BY182
LORANTHACEAE				
Agelanthus	*elegantulus* (Egl.) Polhill & Wiens	Narodenyai	no use	BU9758
Englerina	*woodfordioides* (Schweinf.) Balle	Narodenyai	no use	BY40
MALVACEAE				
Abutilon	*hirtum* (Lam.) Sweet	Sulubei (Sulubei)	1. eaten by livestock, 2. strings for joining sticks, 3. to expel placenta after birth boil root in milk or water	K2008
Abutilon	*longicuspe* A. Rich.	Sulubei (Sulubei)	1. eaten by lifestock, 2. strings for joining sticks, 3. to expel placenta after birth boil root in milk or water	K1986
Abutilon	*mauritianum* (Jacq.) Sweet	Sulubei (Sulubei)	1. eaten by lifestock, 2. strings for joining sticks, 3. to expel placenta after birth boil root in milk/water	K2010, 2013
Hibiscus	*fuscus* Garcke	Lokimekii (Lokimek lo ldonyo)	1. eaten by animals and rarely by livestock, 2. wounds, burn and put ash on wound, 3. strings to join sticks, 4. ceremonial, make protective strings or burn plant and mark body with ash	BY286

Family/Genus	Species	Indigenous name	Use	Collection #
Hibiscus	*ludwigii* Eckl. & Zeyh.	Lkimanshoi	1. eaten by animals and rarely by livestock, 2. wounds, burn and put ash on wound, 3. strings to join sticks, 4. ceremonial, make protective strings or burn plant and mark body with ash	B29
Hibiscus	sp.	Lkimanshoi	1. eaten by animals and rarely by livestock, 2. wounds, burn and put ash on wound, 3. strings to join sticks, 4. ceremonial, make protective strings or burn plant and mark body with ash	B130
Hibiscus	*vitifolius* L.	Lkimanshoi (Nakodonyyo)	1. eaten by animals and rarely by livestock, 2. wounds, burn and put ash on wound, 3. strings to join sticks, 4. ceremonial, make protective strings or burn plant and mark body with ash	K2009
Pavonia	*kilimandscharica* Guerke	Lurabarara	no use	K2011
Pavonia	*patens* (Andr.) Chiov.	Lurabarara	no use	BY82
Pavonia	*urens* Cav.	Sabai	no use	BY97
Sida	*rhombifolia* L.	Desiriko	1. eaten by animals and rarely by livestock	K2014
Sida	*tenuicarpa* Vollesen	Desiriko (Lonini)	1. eaten by animals and rarely by livestock	BY167
MELIACEAE				
Turraea	*abyssinica* A. Rich.	Reteti	1. fruit edible, 2. rungus, 3. firewood	BY280
Turraea	*holstii* Guercke	Reteti	1. fruit edible, 2. rungus, 3. firewood	BUSR

Family/Genus	Species	Indigenous name	Use	Collection #
MENISPERMACEAE				
Cocculus	*pendulus* (J.R. Forst. & G. Forst) Diels	unknown	no use	B200
Stephania	*abyssinica* (Quart.-Dill. & A. Rich.) Walp.	Lokeiet (ILoket, Lagit)	1. eaten by livestock	K2101
MIMOSACEAE				
Acacia	*etbaica* Schweinf.	Ijakai (Ljawai, Lcakwai)	1. leaves eaten by livestock, 2. stomach problems, boiled bark and drink, 3. firewood, 4. fences and houses	BY308
Acacia	*hockii* De Wild	Loilailei (Lerai)	1. leaves eaten by livestock, 2. stomach, childbirth, boil root in water and drink, 3. firewood, 4. construction and fences	K1903
Acacia	*senegal* (L.) Wild	Lkerdeedi (Lnerdedi, Lkerdeedi)	1. leaves eaten by livestock, 2. stomach problems, boil bark and drink, also abortion, 3. firewood, 4. construction and fences	BY357
MONIMIACEAE				
Xymalos	*monospora* (Harv.) Warb.	Lukukut (Lkukuti)	1. firewood	BY4; C0.129; I902
MORACEAE				
Ficus	*scasselatii* Pamp. ssp. *scasselatii*	Reteti (Reteti)	1. firewood, 2. headrests, 3. ceremonial, rain prayer, 4. stomach, crush, boil in water and drink, 4. malaria, boil root, drink, 5. eaten by livestock	BY338
Ficus	*vallis-choudae* Delile	Lomukash	no use	BY295
MYRSINACEAE				
Maesa	*lanceolata* Forssk.	unknown	no use	K1952

Family/Genus	Species	Indigenous name	Use	Collection #
Myrsine	*africana* L.	Seketeti (Seketeti)	1. browsed by livestock, 2. malaria, fever, tuberculosis, boil root and drink, 3. strength, boil plant for soup, 4. stomach problems and tapeworms, boil fruit and drink, 5. wounds, boil fruits and apply, 6. children, boil leaves for dysentery, 7. soak seeds and give livestock as anthelmintic	B22; BY237; C0.12
Rapanea	*melanophloeos* (L.) Mez.	Lapiruni	1. firewood	BY2
MYRTACEAE				
Syzygium	*cordatum* Hochst.	Nkilenyei (Nkilenyei, Nkelenyai)	1. strength, boil root or bark in soup	K1944
NYCTAGINACEAE				
Commicarpus	*helenae* (Roem. & Schult.) Meikle	Loiburubeneiyo (Lpurjapurja, Leucin)	1. eaten by livestock, 2. strength, boil root for tea, 3. malaria, boil crushed root, drink, 4. ear-ache, crush root in cold water, put in ear, 5. wounds, crush and put latex on wound	BY310
OCHNACEAE				
Ochna	*insculpta* Sleumer	Laichmi (Lemaloni, Lpilikwa, Mondokorit)	1. firewood	BY268
OLEACEAE				
Chionanthus	*battiscombei* (Hutch.) Stearn	unknown	no use	BUSR
Jasminum	*abyssinicum* Hochst. ex DC.	Lorosoro (Lmerim)	1. eaten by livestock, 2. bark to join sticks, 3. firewood	BY24
Jasminum	*floribundum* R. Br. ex Fresen.	Lminkis	1. eaten by livestock, 2. bark to join sticks, 3. firewood	K1978
Jasminum	*fluminense* Vell. ssp. *holstii* (Gilg.) Turrill	Lminkis	1. eaten by livestock, 2. bark to join sticks, 3. firewood	B129; K1976

Family/Genus	Species	Indigenous name	Use	Collection #
Olea	*capensis* L.	Loliodoi (Loliontoi, Lopurkei, Siampar)	1. leaves eaten by livestock, 2. construction and fences, 3. rungus, sticks and spear handles, 4. firewood, 5. stomach, boil bark in tea, 6. anthelmintic, soak bark in water overnight, drink	BY10, 98
Olea	*europaea* L. ssp. *africana* (Mill.) P. Green	Tomein, Lgerioi (Lorien, Lnerriyii)	1. leaves eaten by animals, 2. rungus and headrests, 3. construction and fences, 4. firewood, 5. anthelmintic, soak bark overnight and drink	A395; BY88; K1962, 1964
Schrebera	*alata* (Hochst.) Welw.	Lkarate (Letoto)	1. firewood, 2. malaria, boil bark and drink	BY292
ONAGRACEAE				
Epilobium	*hirsutum* L.	Losikiria-Njoi	no use	BY316
OPILIACEAE				
Opilia	*amentacea* Roxb.	Lpuken (Lpukenyi)	1. eaten by livestock, 2. fruit edible, 3. fences, 4. firewood	B136
ORCHIDACEAE				
Aerangis	*thomsonii* (Rolfe) Schltr.	Lkaajo (Lkaajo)	no use	BY102
Brownleea	*parviflora* Lindl.	unknown	no use	K2100
Diaphananthe	*rohrii* (Rchb. f.) Summerh.	unknown	no use	BY131
Eulophia	*petersii* Rchb. f.	Ldinca (Ldinca, Ldincai, Ldinja)	no use	BY144
Polystachya	*confusa* Rolfe	Nardeniai (Nardenyai)	no use	BY110, 113, 132, 133
Polystachya	*piersii* P.J. Cribb.	Nardeniai (Nardenyai)	no use	AR709; BY101
Rangaeris	*amaniensis* (Kraenzl.) Summerh.	unknown	no use	BY142
OROBANCHACEAE				
Orobanche	*minor* Sm.	Lorodo	no use	BY31

Family/Genus	Species	Indigenous name	Use	Collection #
OXALIDACEAE				
Oxalis	*corniculata* L.	Lesermede (Ltapipii-lekera)	1. eaten by animals and rarely by livestock	BY163
PIPERACEAE				
Peperomia	*abyssinica* Miq.	Gitagisii	no use	BY67
Piper	*capense* L.	Loisugi, Loiyeti (Lkoniani)	1. eaten by livestock	BY64, 173; K2022
PLUMBAGINACEAE				
Plumbago	*dawei* Rolfe	Lkiriantus (Lneriantus, Lkiriantus)	1. eaten by livestock, 2. malaria, boil root and drink, 3. stomach, boil and drink	K2061
POACEAE				
Agrostis	*schimperana* Steud.	Lanana	1. eaten by livestock	BY228
Andropogon	sp.	Loroturoto	1. eaten by livestock	BY153
Brachypodium	*flexum* Nees	unknown	no use	BUSR
Bromus	*leptoclados* Nees	unknown	no use	C0.116
Chloris	*roxburghiana* Schult.	Logorereki (Leluben)	1. eaten by animals and rarely by livestock	BY299
Cynodon	*nlemfuensis* Vanderyst var. *nlemfuensis*	Lorotoroto (Lesulluteni)	1. eaten by animals and rarely by livestock	BY284
Digitaria	*velutina* (Forssk.) P. Beauv.	Lanana	1. eaten by livestock	BY296
Ehrharta	*erecta* Lam. var. *abyssinica* (Hochst.) Pilger	Lanana	1. eaten by livestock	BY61; K1930
Eleusine	*multiflora* A. Rich.	Sertet (Leoliin, Lerean)	1. eaten by livestock	BY166
Enteropogon	*macrostachyus* K. Schum. ex Engl.	Loisau (Loisau)	1. eaten by livestock, 2. for thatching houses	BY306
Eragrostis	*schweinfurthii* Chiov.	Nyaput (Lnyaput)	1. eaten by livestock	BY195
Eragrostis	*tenuifolia* (A. Rich.) Steud.	Gujita (Rumoto)	1. eaten by livestock	BY89
Exotheca	*abyssinica* (A. Rich.) Andersson	Log'ururiki (Lopi sedi)	1. eaten by livestock	BY211
Leptochloa	*rupestris* C.E. Hubb.	Loikerekera (Loikirikira)	1. eaten by livestock	BY303
Panicum	*deustum* Thunb.	Laraa	1. eaten by livestock	BY302
Panicum	*hymeniochilum* Nees	Lanana	1. eaten by livestock	BY229

Family/Genus	Species	Indigenous name	Use	Collection #
Rhynchelytrum	*repens* (Willd.) C.E. Hubb.	Lanana	1. eaten by animals and rarely by livestock	BY353
Sinarundinaria	*alpina* (K. Schum.) C.S. Chao & Renvioze	Ltieni (Ltiani)	1. leaves eaten by livestock, 2. for strong containers, 3. ceremonial in circumsition	BY53
Sporobolus	*africanus* (Poir.) Robyns & Tournay	Gujit	1. eaten by livestock	BY99
Stipa	*dregeana* Steud. var. *elongata* (Nees) Stapf.	Lokojitorok	1. eaten by livestock	BY267
PODOCARPACEAE				
Podocarpus	*latifolius* (Thunb.) Mirb.	Biribiriti, Lpilpiriti	1. firewood, 2. construction	A392; BY96; C0.128
POLYGALACEAE				
Polygala	*sphenoptera* Fresen.	Segelaite, Tegelet	no use	BY242, 94
POLYGONACEAE				
Polygonum	*amphibium* L.	unknown	no use	BU9787
Polygonum	*setosulum* A. Rich.	Labelechi	1. eaten by animals and rarely by livestock	BY227; K2034, 2035
Rumex	*bequaertii* De Wild.	Lkaramule	1. fruit edible	K2030
Rumex	*steudelii* A. Rich.	Jipilikwa	1. fruit edible	BY63
POLYPODIACEAE				
Loxogramme	*abyssinica* (Baker) M.G. Price	Gusumwan	no use	BY46
Pleopeltis	*macrocarpa* (Bory ex Willd.) Kaulf.	unknown	no use	C0.145
PRIMULACEAE				
Lysimachia	*volkensii* Engl.	Lokumeki (Lokimeki)	1. eaten by livestock, 2. wounds, burn and apply ash	BY15
PROTEACEAE				
Faurea	*saligna* Harv.	Ilgilma	1. firewood	BY190B; K1936
Protea	*caffra* Meisn. ssp. *kilimandscharica* (Engl.) Chisumpa & Brummitt	unknown	no use	C0.127
PTERIDACEAE				
Pteris	*catoptera* Kunze	Naigerigero	no use	BY69
Pteris	*dentata* Forssk.	Naigerigero	no use	B24, 38, 211; C0.143, 0.152
Pteris	*quadriaurita* Retz	Naigerigero	no use	BU9814

Family/Genus	Species	Indigenous name	Use	Collection #
RANUNCULACEAE				
Clematis	*brachiata* Thunb.	Lgipilikwa (Longorlei)	no use	BY55
Clematis	*simensis* Fresen.	unknown	no use	B41; BY320; K2004
Ranunculus	*multifidus* Forssk.	Laiserieni	no use	BY223; K2032, 2033
Thalictrum	*rhynchocarpum* Quart.-Dill. & A. Rich.	Mosai	no use	BY27
RHAMNACEAE				
Rhamnus	*prinoides* L'Herit	Lkinyil (Lkinyil)	1. malaria, fever, boil and drink, 2. snakebite, boil fruit and leaves, drink and put on bite	BY32; K1971
Rhamnus	*staddo* A. Rich.	Lkukulai (Lkukulai)	1. polio, gonorrhea, malaria, colds, boil for soup, drink	K1946
Scutia	*myrtina* (Burm. f.) Kuntz	Dorokoch (Sanankurrui, Sanankurrii)	1. eaten by livestock, 2. fruit edible, 3. roots boiled for soup, 4. firewood	BY174
Ziziphus	*mucronata* Willd. ssp. *mucronata*	Lukukut (Lkukuti)	1. firewood	I898; K1940
RHIZOPHORACEAE				
Cassipourea	*malosana* (Baker) Alston	Mborolaite (Iburlai)	1. leaves eaten by sheep and goats, 2. for containers	BY11
ROSACEAE				
Alchemilla	*cryptantha* A. Rich.	Mosai	no use	BY33
Prunus	*africana* (Hook. f.) Kalkm.	Malan, Lgilima (Lmalaan, Maralal)	1. leaves eaten by livestock, 2. malaria, boil bark for soup, 3. strength, boil bark for soup, 4. firewood	A390
Rubus	*adolfi-friedericii* Engl.	Lkaramule	1. fruit edible	K1983
Rubus	*apetalus* Poir.	Lkaramule	1. fruit edible	BY7; K1984
RUBIACEAE				
Anthospermum	*usambarense* K. Schum.	unknown	no use	K1999
Galiniera	*saxifraga* (Hochst.) Bridson	Lkukute	1. firewood, 2. construction and fences	BY47
Galium	*aparinoides* Forssk.	Nterepenya	no use	BY39
Pavetta	*abyssinica* Fresen. var. *abyssinica*	Borolaiti (Lmartomi)	1. firewood	BY272
Pavetta	*gardeniifolia* A. Rich. var. *gardeniifolia*	Lekurruk (Lmartomi, Lekurruki)	1. firewood	C0.121

Family/Genus	Species	Indigenous name	Use	Collection #
Pentas	*lanceolata* (Forssk.) Deflers	Lobereti	1. eaten by animals and rarely by livestock, 2. toothbrush, 3. firewood, 4. strings to join sticks	BY271
Pentas	*parvifolia* Hiern	Lokurupani	1. eaten by animals and rarely by livestock, 2. toothbrush, 3. firewood, 4. strings to join sticks	BY304; G5213; K2031
Psychotria	*kirkii Hiern* var *tarambassica* (Bremek.) Verdc.	Lukurog'i (Lekurruki)	1. eaten by livestock, 2. malaria, boil for soup, 3. construction and fences, 4. firewood, 5. young branches for arrows	BY241; G5197; K1966, 1967, 1968
Psychotria	*orophila* Petit	Luisugi	1. eaten by livestock, 2. malaria, boil for soup, 3. construction and fences, 4. firewood, 5. young branches for arrows	BY14, K1953
Psydrax	*schimperiana* (A. Rich.) Bridson ssp. *schimperiana*	Lomasei	1. firewood	BY315
Rhytigynia	*uhligii* (K. Schum. & K. Krause) Verdc.	Borolaite	1. leaves eaten by sheep and goats, 2. for containers	BY277
Tarenna	*graveolens* (S. Moore) Bremek. var. *graveolens*	Lmasei (Lekuriki, Lmasei)	1. eaten by livestock, 2. fruit edible, 3. fences, 4. rungus and sticks, 5. firewood	K1955; G5190
Vangueria	*apiculata* K. Schum.	Lkoromosei	1. eaten by animals and rarely by livestock, 2. fruit edible, 3. fences	G5215
Vangueria	*madagascariensis* J.F. Gmel.	Rugumi	1. eaten by animals and rarely by livestock, 2. fruit edible, 3. fences	BY301, 246; K1954
Vangueria	*volkensii* K. Schum. var. *volkensii*	Lugoromusoi	1. eaten by animals and rarely by livestock, 2. fruit edible, 3. fences	BY275, 328
RUTACEAE				
Clausena	*anisata* (Willd.) Benth.	Lorokigei (Lkoromosyoi, Lkoromosyei)	1. toothbrush	A396; BY5; K1993

Family/Genus	Species	Indigenous name	Use	Collection #
Teclea	*nobilis* Delile	Lgelai (Lgilai, Lkilai, Lnilai)	1. eaten by livestock, 2. construction and fences, 3. toothbrush, 4. rungus, 5. firewood, 6. malaria, boil bark and drink, 7. strength, boil leaves for soup	BY42, 172; K1957
Teclea	*simplicifolia* (Engl.) Verdc.	Lgelai (Lgilai, Lkilai, Lnilai)	1. eaten by livestock, 2. construction and fences, 3. toothbrush, 4. rungus, 5. firewood, 6. malaria, boil bark and drink, 7. strength, boil leaves for soup	K1980
Vepris	*samburuensis* Kokwaro	Kampariti	1. liver problems boil bark and drink	BY313
SALVADORACEAE				
Salvadora	*persica* L. var. *persica*	Sokotei (Syokotei, Sokotei)	1. eaten by livestock, 2. fruit edible, 3. malaria, fever, boil root, drink, 4. stomach, boil root, drink, 5. chest problems, boil root, drink, 5. toothbrush, 6. cattle, boil root in water and give to expel placenta	B212
SANTALACEAE				
Osyridicarpos	*schimperianus* A. DC.	Lokii	1. malaria, boil roots and drink, 2. swollen breasts, soak in water, drink	BY297, 351
Osyris	*abyssinica* (Hochst.) A. Rich.	Lolesyai (Losesyai)	1. eaten by livestock, 2. pregnancy, burn and sit in smoke, 3. strength, boil bark for soup, 4. hold smoke close to swollen udder in cows	K2016
Osyris	*lanceolata* Hochst. & Steud.	Lobuokich	1. eaten by livestock, 2. pregnancy, burn and sit in smoke, 3. strength, boil bark for soup, 4. hold smoke to swollen udder in cows	BY185

Family/Genus	Species	Indigenous name	Use	Collection #
SAPINDACEAE				
Allophylus	*abyssinicus* Hochst. & Radkl.	Kukute (Masanabat)	1. eaten by livestock, 2. firewood, 3. construction and fences	BY12
Allophylus	*griseo-tomentosus* Gilg.	Lmasanapat (Masanabat)	1. eaten by livestock, 2. firewood, 3. construction and fences	K1951
Cardiospermum	*halicacabum* L.	Ltugumi (Ltigomi, Ltikom)	1. snakebites, soak root in water, drink, 2. malaria, crush root, boil and drink, 3. polio, drink boiled root and wash body, 4. firewood, 5. fences, 6. eaten by livestock	BY311
Dodonaea	*viscosa* (L.) Jacq.	Lketurai (Lketurai)	1. toothbrush, 2. eaten by livestock, 3. firewood, 4. construction and fences	K1981
SAPOTACEAE				
Manilkara	*mochisia* (Baker) Dubard	unknown	no use	ATG17563; K1947
Mimusops	*kummel* A. DC.	unknown	no use	BY330, 349
SCHIZEACEAE				
Mohria	*vestita* Baker	Lkowadi	no use	BY254
SCROPHULARIACEAE				
Bartsia	*longiflora* Hochst. ex Benth.	unknown	no use	A546
Cycnium	*tenuisectum* (Standl.) O.J. Hansen	Lorotoroto	no use	BY208
Halleria	*lucida* L.	Ntemelete	1. firewood	A536; BY86; K1975
Hebenstretia	*angolensis* Rolfe	Naitagiteg (Letimi)	1. eaten by animals and rarely by livestock	BY162; K2020
Misopates	*orontium* (L.) Raf.	unknown	no use	AR692
Selago	*thomsonii* Rolfe	Songoroi, Lokuri-Nyanto	no use	A553; BY186, 364
Verbascum	*brevipedicellatum* (Engl.) Hub.-Mor.	Peresi, Ntigei	1. eaten by livestock	A560; BY30, 161
Veronica	*anagallis-aquatica* L.	Sagumai	1. eaten by livestock	BY235
SELAGINELLACEAE				
Selaginella	*dregei* (C. Presl.) Hieron.	unknown	no use	Alexander11859

Family/Genus	Species	Indigenous name	Use	Collection #
SIMAROUBACEAE				
Brucea	*antidysenterica* Lam.	Lorubat (Sonkoroi)	1. eaten by goats and sheep, 2. firewood, 3. arrowpoison, 4. malaria, boil bark and drink	B37; BY16
SOLANACEAE				
Solanum	*aculeatissimum* Jacq.	Ntulelei	1. fever, boil root, drink, 2. throat, boil root, gargle, 3. wounds, put juice on wound	BY65; K1987
Solanum	*benderianum* Engl.	Ntulelei	1. fever, boil root, drink, 2. throat, boil root, gargle, 3. wounds, put juice on wound	K2000
Solanum	*incanum* L	Ntulelei (Ltulelei)	1. fever, boil root, drink, 2. throat, boil root, gargle, 3. wounds, put juice on wound	BUSR; BY13, 266; K1988
Solanum	*indicum* L. ssp. *adoense* (Hochst.) Bitter	Ntulelei (Ltulelei)	1. fever, boil root, drink, 2. throat, boil root, gargle, 3. wounds, put juice on wound	K1989
Solanum	*indicum* L. ssp. *grandifrons* Bitter	Ontulelei (Ltulelei)	1. fever, boil root, drink, 2. throat, boil root, gargle, 3. wounds, put juice on wound	BY75
Solanum	*nigrum* L.	Nyanya (Lmomoi)	1. fever, boil root, drink, 2. throat, boil root, gargle, 3. wounds, put juice on wound	B134, BY221
Solanum	*renschii* Vatke	Ntulelei (Nakuele)	1. fever, boil root, drink, 2. throat, boil root, gargle, 3. wounds, put juice on wound	BY305
Solanum	*schumannianum* Dammer	Ntulele	1. fever, boil root, drink, 2. throat, boil root, gargle, 3. wounds, put juice on wound	BUSR
Solanum	*terminale* Forssk.	Ntulele	1. fever, boil root, drink, 2. throat, boil root, gargle, 3. wounds, put juice on wound	BY28

Family/Genus	Species	Indigenous name	Use	Collection #
STERCULIACEAE				
Dombeya	*goetzenii* K. Schum.	Lporokwai (Lporokwai)	1. eaten by animals and rarely by livestock, 2. firewood, 3. spears and sticks, 4. bark for strings, 5. bees	K1941
Dombeya	*rotundifolia* Planch.	Lawoo (Lawwo)	1. eaten by animals and rarely by livestock, 2. firewood, 3. spears and sticks, 4. bark for strings, 5. bees	G5199
THEPYPTERIDACEAE				
Amauropelta	*bergiana* (Schldtl.) Holttum	Naigerigero	no use	BU9799
Amauropelta	*oppositiformis* (C.Chr.) Holttum	Naigerigero	no use	BY22
Stegnogramma	*pozoi* (Lag.) K. Iwats.	Naigerigero	no use	BU9791, 9792; BY48
THYMAELEACEAE				
Gnidia	*glauca* (Fresen.) Gilg.	Dagaboraa	1. arrow poison, boil bark, put on arrow	BY165; C0.122; K2005
Struthiola	*thomsonii* Oliv.	Loisau	1. eaten by livestock	A545; AR706; BY203
TILIACEAE				
Sparrmannia	*ricinocarpa* (Eckl. & Zeyh.) Kuntze	unknown	no use	K2015
URTICACEAE				
Droguetia	*iners* (Forssk.) Schweinf.	Sabai	no use	BUSR
Girardinia	*diversifolia* (Link.) Friis	Sabai	no use	BY282
Laportea	*alatipes* Hook. f.	Sabai	no use	BY3
Parietaria	*debilis* G. Forst.	Lesiriko	1. eaten by animals and rarely by livestock	BY43
Urera	*hypselodendron* (A. Rich.) Wedd.	Lelkelden	no use	BY100
VERBENACEAE				
Clerodendrum	*johnstonii* Oliv.	unknown	no use	K1960

Family/Genus	Species	Indigenous name	Use	Collection #
Clerodendrum	*myricoides* (Hochst.) Vatke	Lumakutukuti (Lmakutukuti)	1. eaten by animals and rarely by livestock, 2. malaria, cold, polio, gonorrhea, boil root and drink, 3. strength, crush root and boil in soup, 4. bone pain, crush root in cold water, drink, 5. pneumonia, boil root in milk, drink, 6. firewood	BY322; G5200
VIOLACEAE				
Viola	*abyssinica* Oliv.	Mosai	no use	BY54; K2102
VISCACEAE				
Viscum	*triflorum* DC.	Narudenyei	1. fruits edible	BY290
Viscum	*tuberculatum* A. Rich.	Narudenyei (Lmekarokeon)	1. fruits edible	BU9749
VITACEAE				
Cyphostemma	*bambuseti* (Gilg & Brandt) Wild. & R.B. Drumm.	Lorodo (Lordo)	1. fruit and leaves eaten, 2. bone pain, boil leaves and eat, 3. tuberculosis, boil leaves, eat	BY285
Cyphostemma	*kilimandscharicum* (Gilg.) Wild. & R.B. Drumm.	Lorodo (Lordo)	1. fruit and leaves eaten, 2. bone pain, boil leaves and eat, 3. tuberculosis, boil leaves, eat	BY49
Rhoicissus	*revoilii* Planch.	Guilenyai (Loribii)	no use	BY293
WOODSIACEAE				
Cystopteris	*diaphanum* (Bory) Blasdell	Naigerigero	no use	BY25; C0.15
Cystopteris	*fragilis* (L.) Bernh.	Naigerigero	no use	BU9795
INDET				
indet.		Loruki	no use	BY198
indet.			no use	BY321
indet.			no use	BY331
indet.			no use	BY332
indet.			no use	BY335
indet.			no use	BY347

Family/Genus	Species	Indigenous name	Use	Collection #
indet.			no use	BY348
indet.			no use	BY352
indet.			no use	BY354
